# Toxicity of Canola-Derived Glucosinolate Degradation Products in Pigs—A Review

**DOI:** 10.3390/ani10122337

**Published:** 2020-12-09

**Authors:** Jung Wook Lee, In Ho Kim, Tofuko Awori Woyengo

**Affiliations:** 1 Department of Animal Science, South Dakota State University, Brookings, SD 57007, USA; jungwook.lee@uky.edu; 2 Department of Animal Resource and Science, Dankook University, Cheonan-si, Chungnam 31116, Korea; inhokim@dankook.ac.kr; 3 Department of Animal Science, Aarhus University, Blichers Allé 20, DK-8830 Tjele, Denmark

**Keywords:** canola-derived glucosinolates, glucosinolate degradation products, pigs, toxicity

## Abstract

**Simple Summary:**

Canola co-products, which are included in swine diets as a source of amino acids, contain glucosinolates that limit the inclusion of these co-products in swine diets. Aliphatic and aromatic glucosinolates are two major canola co-product-derived glucosinolates. Aliphatic glucosinolates include progoitrin and gluconapin, whereas aromatic glucosinolates include 4-hydroxyglucobrassicin. Glucosinolates are non-toxic, but they are degraded into isothiocyanates, thiocyanate, and nitriles. Isothiocyanates produce goitrin, leading to reduced serum tetraiodothyronine concentration; thiocyanates lead to increased hypothyroidism; nitriles result in hepatic hypertrophy and hyperplasia. Canola-derived glucosinolates are degraded by heat during feed processing, in stomach acid (in the presence of iron), and by myrosinase in various sections of the gastrointestinal tract. Myrosinase is heat-labile and hence most of the myrosinase in canola co-products is inactivated during oil extraction. Notably, microorganisms are highly concentrated in the hindgut of pigs. Thus, the stomach and hindgut are the major sites of glucosinolate degradation in pigs. Most of the glucosinolates that escape degradation by acid in the stomach are degraded in the lower parts of the gastrointestinal tract. Practical swine diets contain iron; hence, degradation of glucosinolates in the stomach may not be limited by iron and may not be easily modified through changes in diet composition. Since the hindgut pH can be modified by diets fed to pigs, the composition of glucosinolate degradation products in the hindgut can be modified through diet modification. A reduction in hindgut pH of pigs due to dietary inclusion of highly fermentable dietary fiber can potentially favor the production of less toxic glucosinolate degradation products derived from canola co-products.

**Abstract:**

Canola co-products are widely included in swine diets as sources of proteins. However, inclusion of canola co-products in diets for pigs is limited by toxicity of glucosinolate degradation products. Aliphatic and aromatic glucosinolates are two major classes of glucosinolates. Glucosinolate degradation products derived from aliphatic glucosinolates (progoitrin) include crambene, epithionitriles, and goitrin, whereas indole-3-acetonitrile, thiocyanate, and indole-3-carbinol are the major aromatic glucosinolates (glucobrassicin)-derived degradation products. At acidic pH (<5.7), progoitrin is degraded by myrosinases to crambene and epithionitriles in the presence of iron, regardless of the presence of epithiospecifier protein (ESP), whereas progoitrin is degraded by myrosinases to goitrin in the absence of ESP, regardless of the presence of iron at neutral pH (6.5). Indole-3-acetonitrile is the major degradation product derived from glucobrassicin in the absence of ESP, regardless of the presence of iron at acidic pH (<4.0), whereas thiocyanate and indole-3-carbinol are the major glucobrassicin-derived degradation products in the absence of ESP, regardless of the presence of iron at neutral pH (7.0). In conclusion, the composition of glucosinolate degradation products is affected by parent glucosinolate composition and hindgut pH. Thus, toxicity of canola co-product-derived glucosinolates can be potentially alleviated by modifying the hindgut pH of pigs.

## 1. Introduction

Canola co-products are the second most commonly used source of amino acids (AAs) in swine diets after soybean meal (SBM) [1]. However, the inclusion of canola co-products in diets for pigs can be limited by their high content of glucosinolates [2]. Canola co-product-derived glucosinolates can be classified into two major groups: aliphatic and aromatic glucosinolates, depending on the AAs that participate in the glucosinolate biosynthesis [3,4]. The dominant aliphatic and aromatic glucosinolates present in canola co-products are progoitrin and gluconapin, and 4-hydroxyglucobrassicin, respectively [5]. Glucosinolates are non-toxic, whereas their degradation products including isothiocyanates, thiocyanate, and nitriles are toxic. For instance, progoitrin-derived isothiocyanates produce goitrin [6], which impairs iodine uptake by the thyroid gland [7], leading to reduced serum tetraiodothyronine (T4) concentration in pigs. Glucobrassicin-derived thiocyanates interfere with iodine uptake by the thyroid gland, leading to reduced synthesis of thyroid hormones and hence increased hypothyroidism [7]. Nitriles result in increased hepatic metabolic activity of various antioxidant and detoxification enzymes [8], leading to increased hepatic hypertrophy and hyperplasia [9]. Glucosinolates found in canola co-products are degraded to toxic metabolites by (1) heat during the process of oil extraction from canola seeds [2,10], (2) dietary myrosinases present in canola co-products [11], (3) acid (in the presence of iron) in the stomach [12], and (4) myrosinases present in various sections of the gastrointestinal tract (GIT) [2,13]. The major sources for the myrosinases are canola co-products (diet) and microorganisms that reside in the GIT. Most of the myrosinases in canola co-products are inactivated during oil extraction since they are heat-labile [14,15]. In pigs, microorganisms are highly concentrated in the hindgut [16]. Thus, the major sites of glucosinolate degradation in pigs are the stomach and hindgut; most of the glucosinolates that escape the degradation by the acid in the stomach are degraded in the lower parts of the GIT.

The toxicity of glucosinolates varies depending on the composition of their degradation products in the GIT [17]. The composition of glucosinolate degradation products is dependent on various factors including the pH of the incubation medium and the presence of epithiospecifier protein (ESP; a non-catalytic cofactor of myrosinase) and ferrous ions in the incubation medium [4,18]. Of these factors, the pH (of the GIT) is the major factor that affects the composition of glucosinolate degradation products in the GIT of pigs fed practical diets because of the following two reasons: First, the ESP is susceptible to heat (is inactivated by heat at 60 °C) [19], and thus, the ESP present in canola seed is inactivated during oil extraction. Second, ferrous ions are available in the GIT of pigs because feedstuffs used to formulate practical swine diets contain iron [20], and swine diets are supplemented with iron-containing mineral premixes to meet iron requirements. Thus, it is apparent that hindgut pH is the major factor that affects the composition of glucosinolate degradation products, and the toxicity of glucosinolates of a given canola co-product can be potentially alleviated by modification of the hindgut pH of pigs. The objective of this paper was to review glucosinolates present in canola co-products and various factors that affect the composition of canola-derived glucosinolate degradation products. In addition, a nutritional strategy that can contribute towards the optimization of utilization of canola co-products in diets for pigs is further discussed.

## 2. Canola

Canola is an oilseed crop of the *Brassica* family, which was developed from rapeseed through breeding [1,13]. Canola has a low content of glucosinolates (less than 30 µmol/g), and its oil has a low content of erucic acid (less than 2%) [21]. The majority of rapeseed grown in Europe have low erucic acid and glucosinolates (known as double-zero rapeseed), whereas a small percentage of high-erucic acid type rapeseed is grown for industrial uses [22]. Various species of canola including *Brassica napus*, *Brassica juncea*, and *Brassica rapa* are grown for the production of oil. Of these species of canola, *B. napus* is the most widely cultivated canola species in North America and Australia [1], whereas *B. juncea* is a new species of canola that was genetically developed to grow well in drier environments of North America [23]. The *B. rapa* was cultivated in northern Europe and Asia. Oil is extracted from canola seeds mainly for human food consumption and the biofuel industry. After canola seed oil extraction, the resulting meals (canola co-products) are available for livestock feeding. Thus, it is of great importance to optimize the utilization of canola co-products in diets fed to swine.

## 3. Canola Co-Products

There are three major methods of extracting oil from canola seeds: solvent extraction, expeller pressing, and cold pressing [24]. When canola oil is solvent-extracted, canola seeds are flaked and steam-heated up to 85 °C for 20 to 40 min to rupture oil-containing cells present in canola seeds. The cooked seeds are screw-pressed to release some oil, and solvent-extracted using hexane to remove most of the remaining oil, and then further desolventized and toasted after oil extraction [24,25,26]. During expeller pressing, canola seeds are also flaked and screw-pressed as previously described for the solvent extraction method, however, the seeds are not solvent-extracted using hexane, and hence the resulting meal is not desolventized and toasted [24]. The cold pressing method is the same as expeller pressing except that the seeds are not cooked prior to pressing and relatively less pressure is applied during oil extraction by the cold pressing method compared to that of the expeller pressing method to ensure that the meal temperature is maintained at approximately 50 to 60 °C [27]. Thus, of these three methods of oil extraction, solvent extraction is the most efficient, followed by expeller pressing, and then cold pressing. Cold pressing is a relatively new method of oil extraction, which results in the production of chemical-free oil [27]. Solvent extraction and expeller pressing are the most widely utilized methods for oil extraction [24,27]. Solvent extraction, expeller pressing, and cold pressing result in the production of solvent-extracted canola meal (SECM), expeller-pressed canola meal (EPCM), and cold-pressed canola expellers (CPCE), respectively, which are available for livestock feeding. In North America, the solvent extraction method is the most conventional method of extracting oil from canola seeds, and hence SECM is the most commercially available canola co-product. However, CPCE is increasingly becoming available for livestock feeding, mainly due to rising demand for natural oil products over the past few years [28]. In addition, small-scale extractors that are used to extract oil by the cold pressing method are relatively cheap. In addition to rising demand and inexpensive production cost, it is environmentally safer to obtain oil from canola seeds using the cold pressing method than the solvent extraction because the cold pressing method does not involve thermal or chemical treatment.

Nutrient composition and energy values of canola co-products and conventional solvent-extracted soybean meal are presented in Table 1. Canola co-products contain less crude protein (CP)and hence less indispensable and dispensable AA, but greater ether extract (EE), acid detergent fiber (ADF), and neutral detergent fiber (NDF) than conventional soybean meal. Within the canola co-products, CPCE contains less CP, ADF, and NDF, but more EE than that of SECM and EPCM. The greater EE content for CPCE than that for the other canola co-products is due to the fact that cold pressing is a less efficient method of oil extraction than solvent extraction or expeller pressing; the lower NDF content of CPCE than that of the other canola co-products is attributed to CPCE with a high content of residual oil [12,24], which dilutes the concentration of other components in the CPCE. The digestible energy (DE) and net energy (NE) values for CPCE are highest, followed by EPCM and then SECM. The greater DE and NE values for CPCE than those for EPCM or SECM are attributed to higher residual oil content and lower fiber content in CPCE compared to those in the other canola co-products. Thus, nutrient composition, energy digestibility, and energy values of canola co-products can vary depending on the oil extraction method. Furthermore, CPCE is a greater energy source than are SECM or EPCM due to its high residual oil and low fiber content.

## 4. Glucosinolates

Glucosinolates are secondary plant metabolites, which are composed of β-D-thioglucose, a sulphonated oxime, and side-chain groups [4]. Glucosinolates are biosynthesized in three major steps: side chain elongation (AA chain elongation), glucone biosynthesis (glucosinolate biosynthesis from AAs), and side chain modification (modification for side chains of glucosinolates) [17]. Two major chemical groups of glucosinolates include aliphatic and aromatic glucosinolates [4]. Aliphatic glucosinolates are biosynthesized from alanine, isoleucine, leucine, methionine, or valine, whereas aromatic glucosinolates are derived from tyrosine, phenylalanine, or tryptophan [31,32]. In *B. napus* canola co-products, progoitrin and gluconapin are the major aliphatic glucosinolates, whereas 4-hydroxyglucobrassicin is the dominant aromatic glucosinolates. The major aliphatic and aromatic glucosinolates present in *B. juncea* co-products are gluconapin and 4-hydroxyglucobrassicin [5]. Toxicity of degradation products that are derived from aliphatic glucosinolates is greater than the toxicity of those derived from aromatic glucosinolates [33,34].

Total, aliphatic, and aromatic glucosinolate contents in canola co-products are presented in Table 2. Of these canola co-products, *B. juncea* co-products have a greater content of total and aliphatic glucosinolates, but a lower content of aromatic glucosinolates than *B. napus* canola co-products. Within *B. napus* canola co-products, SECM contains less total glucosinolates than EPCM or CPCE. The lower total glucosinolate content in SECM than that in EPCM or CPCE is attributed to the fact that SECM is desolventized and toasted [10], whereas EPCM or CPCE are not subjected to desolventization and toasting during the process of oil extraction [24]. The SECM is subjected to heat during the desolventization and toasting process, leading to degradation of heat-labile glucosinolates. The lower aromatic glucosinolate content in SECM than that in EPCM or CPCE is due to the fact that aromatic glucosinolates are more degradable by heat than are aliphatic glucosinolates [10]. Indeed, the reduction in the aromatic glucosinolate content was greater than that in the aliphatic glucosinolate content in rapeseed meal due to toasting [35]. The differences between SECM and EPCM or CPCE with regard to the aromatic glucosinolate content can be explained by the fact that the SECM is exposed to more heat than is the EPCM or CPCE during the process of oil extraction. Thus, it is apparent that total and individual content of glucosinolates in canola co-products are dependent on canola species and oil extraction process. The negative effects of glucosinolates on growth performance and organ weights of pigs are discussed in the following sections.

## 5. Canola Co-Product Utilization in Swine Diets

### 5.1. Effects of Purified Glucosinolates on Growth Performance of Pigs

The effects of increasing dietary levels of glucosinolates through dietary inclusion of canola co-products on growth performance and voluntary feed intake of pigs were determined in several previous studies [29,36,37,38,40,42,43,45,46,47,49] and reviewed [2]. In summary, an increase in the dietary level of *B. napus* canola co-products-derived glucosinolates to 2.22 µmol/g did not affect body weight (BW) gain and voluntary feed intake of pigs, whereas increasing the level of total glucosinolates to ≥2.75 µmol/g in diets through dietary inclusion of *B. napus* canola co-products reduced BW gain and voluntary feed intake of pigs. In addition, an increase in dietary level of Juncea canola co-products-derived glucosinolates from 0 to 2.60 µmol/g reduced BW gain and voluntary feed intake of pigs. The effects of adding purified glucosinolates in diets for animals on growth performance have also been investigated. Bille et al. (1983) reported a 22 and 23% reduction in body weight gain and feed intake of growing rats, respectively, due to the consumption of a diet with purified progoitrin at 5 mg/g for 5 d [50]. Similarly, Bjerg et al. (1989) observed a 7 and 6% reduction in weight gain and feed intake of rats, respectively, in 10 d due to an increase in the level of total glucosinolates from 0 to 12.5 µmol/g through dietary inclusion of pure progoitrin [51]. However, Vermorel et al. (1986) reported unaffected growth performance of growing rats over 29 d due to the addition of pure glucobrassicin to the diet at 0.5 g/kg [34], which is attributed to the fact that glucobrassicin is less toxic than progoitrin. From these studies, it is apparent that glucosinolates reduce growth performance of animals, and that an increase in the amount of glucosinolates in diets for pigs to the level above 2.60 µmol/g through dietary inclusion of *B. napus* canola co-products negatively affects BW gain and feed intake. However, the tolerance level of glucosinolates derived from Juncea SECM is less than that derived from Napus SECM by pigs, which can be partly explained by the greater content of aliphatic glucosinolates in Juncea SECM than in Napus SECM. Thus, further research is warranted to determine the optimal level of Juncea canola-derived glucosinolates in diets for pigs without reducing growth performance.

### 5.2. Effects of Purified Glucosinolates on Organ Weights of Pigs

The effects of increasing amounts of glucosinolates in diets for pigs through dietary inclusion of canola co-products on organ weights and thyroid hormones were determined in previous studies [36,40,43,52] and reviewed [2]. In summary, a dietary level of canola-derived glucosinolates less than 2.50 µmol/g did not affect liver and thyroid gland weights of pigs. Results from previous studies on the effects of including purified glucosinolates on organ weights have been reported. Bille et al. (1983) reported a 6, 20, and 110% increase in liver, kidneys, and thyroid gland weights of growing rats, respectively, due to the consumption of diets with purified progoitrin at 5 mg/g for 5 d [50]. Bjerg et al. (1989) reported a 4% increase in liver weight of rats during a 10-day feeding period due to an increase in the level of total glucosinolates from 0 to 12.5 µmol/g through dietary inclusion of purified progoitrin [51]. Vermorel et al. (1986) similarly reported a 17, 9, and 34% increase in liver, kidneys, and thyroid gland weights of growing rats, respectively, during a 29-day feeding period due to the addition of purified progoitrin to the diet at 3 g/kg [34]. However, in the same study, the addition of pure glucobrassicin to the diet at 0.5 g/kg did not affect organ weights of growing rats, likely because glucobrassicin is less toxic than is progoitrin. This increase in liver, kidney, and thyroid gland weights of pigs fed canola co-products-containing diets can be explained by the adverse effects of certain glucosinolates in the canola co-products on these organs. From the pig studies, it appears that a dietary level of canola-derived glucosinolates that is less than 2.10 µmol/g does not reduce liver and thyroid gland weights relative to live BW, synthesis of thyroid hormones, nor thyroid hormone levels in serum.

An increase in visceral organ weight is positively associated with an increase in metabolic activity in the same organ [53]. An increase in metabolic activities in visceral organs results in increased energy expenditure by these organs at the expense of skeletal tissue deposition [54]. Thus, the increase in organ weights of pigs due to dietary inclusion of *B. napus* canola co-products indicates increased utilization of dietary energy for the maintenance of these organs. Additionally, thyroid hormones are involved in the regulation of energy metabolism with the body, implying that the reduction in thyroid hormone synthesis negatively affects the growth and development of animals [55,56]. Thus, the increased organ weights and reduced thyroid hormone levels due to increasing dietary levels of canola co-products-derived glucosinolates can result in a reduction in the growth and development of pigs.

## 6. Glucosinolate Degradation Products

Glucosinolates are present in cellular compartments of plants and are physically segregated from the myrosinase (β-thioglucosidase glucohydrolase; EC 3.2.3.1) that is localized in the cytoplasm of myrosin cells [57]. Upon ingestion and disruption of plant tissues, glucosinolates are hydrolyzed by the myrosinase present in canola co-products (dietary myrosinase) [4]. Gastrointestinal tract microorganisms also produce myrosinase that can degrade ingested glucosinolates [13]. Glucosinolates are heat-labile and thus they can additionally be degraded by heat during the process of oil extraction from canola seeds [10,58]. Finally, glucosinolates can be degraded nonenzymatically under acidic conditions [59]. For instance, at the acidic pH found in the stomach of pigs, glucosinolates are degraded to nitriles, which are absorbed in the stomach and upper part of the small intestine regardless of the presence of myrosinases [12]. Thus, dietary glucosinolates can be degraded to toxic metabolites by (1) dietary myrosinase when myrosin cells are disrupted during ingestion or oil extraction (enzymatic degradation), (2) non-enzymatic degradation of glucosinolates under acid conditions (chemical degradation), (3) microbial myrosinase in the lower part of the GIT (microbial degradation), and (4) heat that the seeds and meal are subjected to during oil extraction processes (thermal degradation) [2,59].

Intact glucosinolates themselves are non-toxic, but their glucosinolate degradation products are toxic [4]. Of these glucosinolate degradation products, isothiocyanates have been the most widely studied due to their chemoprotective attributes in human nutrition [17]. Isothiocyanates have been shown to induce hepatic phase II detoxification enzymes including glutathione S-transferase and quinone reductase in rats [60,61]. However, isothiocyanates (2-hydroxy-3-butenyl isothiocyanates) that are derived from progoitrin cyclize to produce goitrin [6]. Goitrin interferes with iodine uptake by the thyroid gland [7], leading to reduced serum tetraiodothyronine (T4) concentration in pigs. For instance, dietary goitrin supplementation at 200 mg/kg of diet reduced serum T4 level of rats by 60% [62]. Nishie and Daxenbichler (1980) reported a 12 and 10% increase in liver and thyroid gland weights of rats, respectively, due to oral administration of goitrin at 200 mg/kg BW [33]. Isothiocyanates (indole-3-methyl-isothiocyanates) derived from glucobrassicin are converted to indole-3-carbinol [18]. The indole-3-carbinol is degraded to thiocyanates, which impair iodine uptake by the thyroid gland, leading to reduced synthesis of thyroid hormones and hence increased hypothyroidism [7]. Supplemental thiocyanate at 1000 mg/kg of diet reduced serum T4 of growing pigs by 39% [63]. Nitriles increase the hepatic metabolic activity of various antioxidant and detoxification enzymes in mice and lesions of the kidneys in rats [8], leading to hepatic hypertrophy and hyperplasia [9]. However, nitriles (crambene) derived from progoitrin were also shown to induce glutathione S-transferase and quinone reductase [64], which are chemoprotective, implying that progoitrin-derived nitriles are health-promoting metabolites. The severity of the toxicity of dietary glucosinolates in canola co-products can vary depending on the composition of their resulting glucosinolate degradation products. Thus, it is of great importance to determine various factors that affect the composition of glucosinolates degradation products in order to develop strategies to optimize utilization of canola co-products in diets for pigs.

## 7. Factors Affecting Composition of Degradation Products of Glucosinolates

As previously mentioned, glucosinolates are not toxic, but their degradation products are toxic depending on their composition. The composition of glucosinolate degradation products is dependent on several factors including the composition of the parent glucosinolates, pH of the reaction medium, and the presence or absence of ESP and iron in the reaction medium. These factors are discussed and summarized in Table 3.

### 7.1. Degradation Products of Progoitrin at Acidic and Neutral pH

The two major glucosinolate degradation products that are derived from progoitrin include goitrin (5-vinyl-1,3-oxazolidine-2-thione; cyclized isothiocyanate) and nitriles including crambene (1-cyano-2-hydroxy-3-butene) and epithionitrile (1-cyano-2-hydroxy-3,4-epithiobutane [65,66]. There are various forms of nitriles including crambene and epithionitriles. Progoitrin is degraded by myrosinases to the nitrile crambene at acidic pH in the presence of iron but in the absence of ESP. For instance, Macleod and Rossiter (1987) reported in vitro production of crambene from *B. napus* rapeseed progoitrin in an acidic (pH of 5.7) incubation medium that contained iron, but lacked ESP [67]. Matusheski et al. (2006) observed in vitro production of crambene from broccoli progoitrin at the expense of goitrin in an acidic (pH of 5.5) incubation medium that contained iron but lacked ESP [66]. Similarly, Frandsen et al. (2019) observed in vitro production of crambene from yellow mustard progoitrin in an acidic (pH of 3.0 or 5.0) incubation medium that contained iron but lacked ESP [12]. Leoni et al. (1993) reported in vitro production of crambene from Crambe abyssinica seeds progoitrin in an acidic (pH of 5.0) incubation medium that contained iron but lacked ESP [68]. In the presence of both iron and ESP, progoitrin is degraded by myrosinases to both crambene and epithionitriles at acidic pH conditions. For instance, Galletti et al. (2001) observed in vitro production of crambene and epithionitriles from progoitrin in *Crambe abyssinica* seeds in an acidic (pH of 5.0) incubation medium that contained both iron and ESP [6]. Similarly, Matusheski et al. (2006) reported in vitro production of crambene and epithionitriles from broccoli progoitrin at the expense of goitrin in acidic pH (pH of 5.5) incubation medium that contained both iron and ESP [66].

At neutral pH in the presence of iron but in the absence of ESP, progoitrin is degraded by myrosinases to goitrin. For instance, Leoni et al. (1994) observed in vitro production of goitrin from the progoitrin of *Crambe abyssinica* seeds in an incubation medium (that contained iron but lacked ESP) with neutral pH (pH of 6.5) [69]. Daubos et al. (1998) observed in vitro production of goitrin from the progoitrin of *Crambe abyssinic* meal in an incubation medium (that contained iron but lacked ESP) with neutral pH (pH of 6.5) [70]. Progoitrin is degraded by myrosinases to goitrin at neutral pH in the absence of both iron and ESP. For instance, Galletti et al. (2001) reported in vitro production of goitrin from the progoitrin of *Crambe abyssinica* seeds in an incubation medium (that lacked both iron and ESP) with neutral pH (pH of 6.5) [6]. Xie et al. (2011) observed in vitro production of goitrin from *Radix isatidis* progoitrin in an incubation medium (that lacked both iron and ESP) with neutral pH (pH of 6.5) [71].

### 7.2. Degradation Products of Glucobrassicin at Acidic and Neutral pH

The indole-3-acetonitrile, indole-3-carbinol, and thiocyanate are the three major degradation products that are derived from glucobrassicin. Glucobrassicin is degraded by myrosinases to indole-3-acetonitrile at acidic pH in the presence of iron but the absence of ESP. For instance, Agerbirk et al. (1998) reported increased in vitro production of indole-3-acetonitrile from broccoli glucobrassicin due to a decrease in the pH of the incubation medium (that contained iron but lacked ESP) from pH 7.0 to 4.0 [18]. Bradfield and Bjeldanes (1987) reported increased in vitro production of indole-3-acetonitrile from *Brassica oleracea*-derived glucobrassicin due to a decrease in the pH of the incubation medium (that lacked both iron and ESP) from pH 5.0 to 3.0 [72]. Latxague et al. (1991) observed increased in vitro production of indole-3-acetonitrile from synthetic glucobrassicin at the expense of indole-3-carbinol or thiocyanate in an incubation medium (that lacked both iron and ESP) with acidic pH (pH of 3.0) [73]. Similarly, Chevolleau et al. (1997) reported increased in vitro production of indole-3-acetonitrile from synthetic glucobrassicin at the expense of thiocyanate in an incubation medium (that lacked both iron and ESP) with acidic pH (pH of 3.0) [74].

In the presence of iron but the absence of ESP, glucobrassicin is degraded by myrosinases to thiocyanate at neutral pH. For instance, Agerbirk et al. (1998) reported increased in vitro production of thiocyanate from broccoli glucobrassicin due to an increase in the pH of the incubation medium (that contained iron but lacked ESP) from pH 4.0 to 7.0 [18]. Glucobrassicin is degraded by myrosinases to indole-3-carbinol at neutral pH in the absence of both iron and ESP. For instance, Bradfield and Bjeldanes (1987) observed increased in vitro production of indole-3-carbinol from *Brassica oleracea* glucobrassicin due to an increase in the pH of the incubation medium (that lacked both iron and ESP) from pH 5.0 to 7.0 [72]. Chevolleau et al. (1997) reported increased in vitro production of indole-3-carbinol from synthetic glucobrassicin in an incubation medium (that lacked both iron and ESP) with neutral pH (pH of 7.0) [74].

### 7.3. Various Factors Affecting Degradation of Glucosinolates

Heat treatment results in increased thermal degradation of glucosinolates to nitriles. For instance, Slominski and Campbell (1989) reported increased in vitro production of indole-3-acetonitrile from cabbage glucobrassicin due to heat treatment at 100 °C for 50 min [75]. Slominski and Campbell (1989) observed increased in vitro production of indole-3-acetonitrile from rapeseed meal glucobrassicin due to heat treatment at 100 °C for 5 min [76]. Similarly, Hanschen et al. (2012) reported increased in vitro production of nitriles from aliphatic glucosinolates in broccoli due to heat treatment at 100 °C that was continued for 60 min, implying that heat treatment results in increased production of nitriles from glucosinolates during the process of oil extraction [58]. Thus, SECM is expected to have a greater content of thermally induced glucosinolate degradation products (nitriles) than that of CPCE because it is subjected to more heat than the latter.

Most of the ESP and myrosinases present in canola co-products especially SECM and EPCM are denatured by heat during the production of the co-products from canola seeds [77] because, like most other bioactive proteins, they are heat-labile [14,15,66]. For instance, Ludikhuyze et al. (1999) observed a reduction in myrosinase activity in broccoli by greater than 95% due to its heat treatment at 60 °C for 20 min [14]. Eylen et al. (2007) reported myrosinase inactivation due to its heat treatment at 60 °C for 10 min [15]. Similarly, Matusheski et al. (2004) observed complete ESP inactivation due to heat treatment at ≥50 °C for 10 min [19]. The minimum temperature to which SECM and EPCM are exposed to during their production is 103 °C [26], whereas the minimum temperature to which CPCE is exposed during its production is 50 °C [27]. In addition, iron is present in the GIT of pigs [20] because iron supplements are added in practical swine diets to meet iron requirements. Iron and ESP are not the major factors that affect the composition of glucosinolate degradation products of the canola co-products. Thus, it is apparent that the parent glucosinolate composition and hindgut pH are the major factors that affect the composition of glucosinolate degradation products. Since the composition of parent glucosinolates in canola co-products vary depending on canola species and oil extraction method, the toxicity of glucosinolates of a given canola co-product can be potentially alleviated by modification of the hindgut pH of pigs.

**Table 3:** Effects of various factors on the composition of glucosinolate degradation products derived from progoitrin and glucobrassicin.

Item	Parent Glucosinolates	Incubation (Reaction) Medium			Degradation Products	References
		pH	Iron	ESP ^1^		
Rapeseed (Napus)	Progoitrin	5.7	+	-	Crambene	[67]
Broccoli	Progoitrin	5.5	+	-	Cramen	[66]
Yellow mustard	Progoitrin	3.0 or 5.0	+	-	Crambene	[12]
*Crambe abyssinica* seeds	Progoitrin	5.0	+	-	Crambene	[68]
*Crambe abyssinica* seeds	Progoitrin	5.0	+	+	Crambene and epithionitriles	[6]
Broccoli	Progoitrin	5.5	+	+	Crambene and epithionitriles	[66]
*Crambe abyssinica* seeds	Progoitrin	6.5	+	-	Goitrin	[69]
*Crambe abyssinic* meal	Progoitrin	6.5	+	-	Goitrin	[70]
*Crambe abyssinica* seeds	Progoitrin	6.5	-	-	Goitrin	[6]
*Radix isatidis*	Progoitrin	6.5	-	-	Goitrin	[71]
Broccoli	Glucobrassicin	4.0	+	-	Indole-3-acetonitrile	[18]
*Brassica oleracea*	Glucobrassicin	3.0	-	-	Indole-3-acetonitrile	[72]
Synthetic	Glucobrassicin	3.0	-	-	Indole-3-acetonitrile	[73]
Synthetic	Glucobrassicin	3.0	-	-	Indole-3-acetonitrile	[74]
Broccoli	Glucobrassicin	7.0	+	-	Thiocyanate	[18]
*Brassica oleracea*	Glucobrassicin	7.0	-	-	Indole-3-carbinol	[72]
Synthetic	Glucobrassicin	7.0	-	-	Indole-3-carbinol	[74]

^1^ ESP = epithiospecifier protein.

## 8. Reduction of Hindgut pH of Pigs

Fermentable dietary fiber is poorly digested in the small intestine but highly fermented in the large intestine of pigs, leading to increased production of volatile fatty acids (VFA) and hence reduced hindgut pH of pigs [78]. Thus, fermentable dietary fiber can potentially be included in swine diets to reduce the toxicity of glucosinolate degradation products. Highly fermentable dietary fiber includes inulin and resistant starch. For instance, Ten Bruggencate et al. (2004) reported a 17% decrease in cecal pH of rats due to dietary inclusion of inulin at 60 g/kg [79], whereas Bird et al. (2007) reported a 17, 20, 22, and 21% decreases in pH of the cecum and proximal, mid, and distal colon of growing pigs, respectively, due to dietary inclusion of high amylose (HA)-starch (resistant starch) at 515 g/kg [78]. Fouhse et al. (2015) observed 22 and 20% reductions in cecal and colonic pH of nursery pigs, respectively, due to dietary inclusion of HA-starch at 67% [80]. Lee et al. (2020) reported 4, 9, 9, and 6% reductions in the pH of the cecum and proximal, mid, and distal colon of nursery pigs, respectively, due to dietary inclusion of HA-starch (resistant starch) and demonstrated that the negative effects of dietary CPCE on thyroid gland functions of nursery pigs can be alleviated by dietary HA-starch [81]. Feedstuffs that contain resistant starch are cheaper and more available in large quantities than other sources of highly fermentable dietary fiber such as inulin, and thus they can be a good source of highly fermentable dietary fiber in canola co-products-based diets for pigs.

## 9. Conclusions

Progoitrin and glucobrassicin are the major aliphatic and aromatic glucosinolates, respectively, present in canola co-products fed to pigs. The toxicity of canola-derived glucosinolates is dependent on the composition of their degradation products in the GIT of pigs. The composition of degradation products derived from glucosinolates varies depending on the pH of the incubation medium, the presence or absence of ESP, and ferrous ions in the incubation medium. The pH of the incubation medium (the GIT) is the major component affecting the composition of glucosinolates-derived degradation products in pigs. Low pH in the hindgut of pigs favors the production of fewer toxic metabolites from glucosinolates, implying that the tolerable level of glucosinolates by pigs and hence a dietary level of canola co-products can be increased by reducing the hindgut pH. The hindgut pH can be reduced by dietary inclusion of highly fermentable dietary fiber including inulin and resistant starch, which can potentially alleviate the toxicity of canola-derived glucosinolates and optimize the utilization of canola co-products in diets for pigs. Since information with regard to the effects of reducing hindgut pH on the toxicity of glucosinolate degradation products in pigs is limited, further research is warranted to investigate the effects of low hindgut pH on the composition of glucosinolate degradation products in pigs fed canola co-products-based diets.

## Figures and Tables

**Table 1:** Nutrient composition and energy values of canola co-products (% dry matter basis) ^1^.

Feedstuff ^2^	Content, %					Energy, kcal/kg	
	Moisture	CP	EE	NDF	ADF	DE	NE
SECM ^3^	8.67	41.1	3.53	24.8	16.9	3584	2069
EPCM ^3^	6.89	37.8	10.7	25.5	18.9	4059	2525
CPCE ^4^	12.7	29.6	23.1	17.6	13.1	5080	3550
SBM ^3^	10.0	53.6	1.71	9.23	5.93	4067	2345

^1^ Values for digestible energy and net energy (kcal/kg) were adapted from a previous study and the NRC (2012) [29,30]. ^2^ SECM = solvent-extracted canola meal, EPCM = expeller-pressed canola meal, CPCE = cold-pressed canola expellers, SBM = soybean meal. ^3^ Values for EPCM were adapted from [30]. ^4^ Values for CPCE were adapted from a previous study [11].

**Table 2:** Total, aliphatic, and aromatic glucosinolate contents in canola co-products for pigs ^1^.

Item	Glucosinolate Content, µmol/g		References
	Mean	Range	
Total glucosinolates			
*B. napus* SECM	6.02	3.70–8.60	[10,29,36,37,38,39,40]
*B. napus* EPCM	10.7	9.27–15.3	[10,29,37,41,42,43]
*B. napus* CPCE	11.9	11.1–12.7	[44,45]
*B. juncea* SECM	13.9	10.8–17.2	[5,39,46]
Aliphatic glucosinolates			
*B. napus* SECM	4.86	3.02–6.16	[10,37,39,40]
*B. napus* EPCM	7.66	4.85–10.6	[10,41,43,47]
*B. napus* CPCE	4.00	3.95–4.05	[44,45]
*B. juncea* SECM	12.1	10.4–14.1	[5,39,46]
Aromatic glucosinolates			
*B. napus* SECM	1.53	0.82–2.80	[37,39,40,48]
*B. napus* EPCM	3.98	1.73–4.78	[41,43,47,48]
*B. napus* CPCE	7.45	6.69–8.21	[44,45]
*B. juncea* SECM	1.39	0.49–3.10	[5,39,46]

^1^ SECM = solvent-extracted canola meal; EPCM = expeller-pressed canola meal; CPCE = cold-pressed canola expellers.
